# Compliant Mechanism-Based Sensor for Large Strain Measurements Employing Fiber Optics

**DOI:** 10.3390/s22113987

**Published:** 2022-05-24

**Authors:** Oleg Shiryayev, Nader Vahdati, Fook Fah Yap, Haider Butt

**Affiliations:** 1 Department of Mechanical Engineering, ECB 301J, University of Alaska Anchorage, 3211 Providence Dr., Anchorage, AK 99508, USA; 2 Department of Mechanical Engineering, Khalifa University of Science and Technology, Healthcare Engineering Innovation Center, SAN Campus, Abu Dhabi P.O. Box 127788, United Arab Emirates; nader.vahdati@ku.ac.ae (N.V.); haider.butt@ku.ac.ae (H.B.); 3 Department of Mechanical and Aerospace Engineering, Nanyang Technological University, Singapore 639798, Singapore; mffyap@ntu.edu.sg

**Keywords:** compliant mechanisms, optical fiber, strain, Fiber Bragg Gratings, large deformations

## Abstract

We propose a sensor design for measurement of large strains where direct application of a fiber optic strain gauge is impossible due to the stiffness mismatch between the optical fiber and the structure under test. The sensor design is based on a rhombus type compliant mechanism, which functions to attenuate input strain and transfer it to the ends of the sensing beam with the mounted optical strain gauge. We developed an analytical model of the sensor, which allows us to relate actuation forces, input displacement/strain, and output strain. The analytical model was verified with the finite element analysis and validated against an experimental prototype. The prototype sensor was able to handle input strains exceeding ±2.5 × 10^5^ µε. Potential application areas of the proposed sensor include compliant elastomeric structures, wearables, and soft robotics.

## 1. Introduction

In many applications it is necessary to measure strain or displacement on a part that is relatively flexible or made of low modulus material. For example, measurements of deformation on elastomeric mounts used in buildings, aircraft, and machinery, rubber flexures, parts made of polymers, etc. These parts may undergo very large strains, in the order of 10–30% ($0.1-0.3\times 10^{6}$ µε). Conventional resistive foil strain gauges are not suitable for such applications due to the limited strain range (1–5%), and the fact that they also create a stiffening effect by altering the local strain field at the location where they are installed because their modulus is several times higher than that of the test article material [1,2,3,4]. An alternative to foil strain gauges in situations with high strains is to use extensometers, or non-contact techniques, such as digital image correlation systems [5,6,7]. These alternatives work well in many settings, but they are not suitable for applications with difficult to access locations, restricted space, and large distances between the measurement location and the data acquisition and processing equipment.

Fiber optic-based sensors are becoming very popular in a wide variety of applications, including measurement of strain, vibration, temperature, humidity, magnetic fields, and others [8,9,10,11]. In particular, fiber Bragg grating (FBG) sensors have become one of the primary fiber optic measurement technologies [12,13,14,15,16,17]. Their growing popularity is due to several advantages that they possess over traditional sensing technologies: (i) they do not require electrical power, (ii) optical fiber serves as the sensing medium and the signal transmission line, (iii) immunity to electromagnetic interference and ground loops, (iv) capability of being multiplexed and deployed over very long distances, (v) high sensitivity and resolution. FBG-based sensors are used for strain measurement in mechanical, civil, aerospace, and biomedical applications.

Fiber optic sensors also exhibit a stiffening effect on the test article. Considering a typical silica glass optical fiber with a modulus of approximately 70 GPa, and fiber diameter of 125 µm, which is actually larger than those of typical resistance foil strain gauges (10 GPa and 60 µm), the FBG will tend to underestimate the strain due to its higher stiffness compared to a typical resistance strain gauge [18]. Silica glass FBG sensors are also limited by the amount of strain they can be subjected to, which is about 5–10 × 10^3^ µε. An ideal sensor for measurements of large strains on low modulus or low stiffness structures needs to be highly compliant and be able to withstand large strains.

Rubber components are usually designed to be loaded in compression, but tension strains can also occur. Rubber components are generally designed not to exceed tensile strains above 30%, even though some research [19] suggests that microcavity or micro-cracks can occur at tensile strains as low as 17% to 20%. From time to time, strains can reach or exceed 50%, when sudden large motions or shocks are present, but such large strains are rare. Current commercially available strain gages, capable of measuring rubber strains, are typically usable for measuring strains up to 20%.

In this work we develop an FBG-based sensor that incorporates a compliant mechanism, which serves the purpose of attenuating the strain experienced by the structure under test so as to bring it within the typical operating range of FBG sensors, and in addition to that, reducing the stiffening effect and force feedback caused by the sensor itself. Compliant mechanisms rely on elastic deformations of flexible hinges [20] for transferring loads and motions between rigid parts or links [21]. Their advantages over traditional mechanisms include high resolution of motions, absence of friction, low manufacturing costs, and possibility of miniaturization. Their primary uses are in precision positioning and instrumentation via amplification of motions or forces [22,23,24,25,26,27,28].

Several different types of compliant mechanisms have emerged over time: lever type [29,30,31], bridge type [32,33,34], and Scott-Russell type [35,36]. In this work, we employ the rhombus mechanism with elastic flexures, which is related to the bridge type mechanisms. Mechanism geometry that follows topology of a rhombus allows realization of a generous range of amplification/attenuation ratios, while keeping compact dimensions. Integration of compliant mechanism serves the purpose of attenuating displacement and reducing the stiffness that is added onto the test article by the sensor. The FBG strain gauge is attached on the sensing beam connected to the output terminals of the compliant mechanism. The resulting sensor design is suitable for measurement of in-plane, uniaxial deformations along the line, connecting input terminals of the compliant mechanism.

In the following section we present the details of analytical modeling that is used for design purposes and verify it against the finite element model. Then, we present experimental data obtained from evaluation of the prototype sensor and provide some concluding remarks.

## 2. Materials and Methods

**Figure 1:**
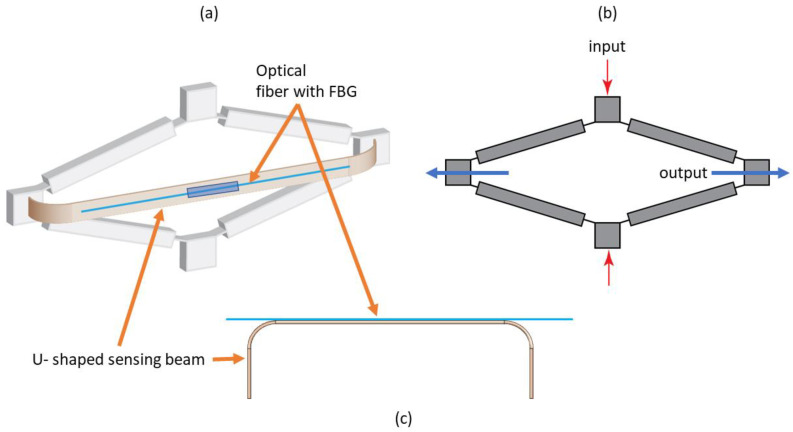
Schematic of the sensor with the U-shaped beam and the rhombus-type mechanism: (**a**) Overall view of the sensor including the compliant mechanism and the sensing beam; (**b**) Plan view of the rhombus mechanism; (**c**) Plan view of the sensing beam and the optical strain sensor.

The sensor design investigated here consists of the rhombus-type compliant mechanism, which transfers the motion and loads onto the elastic U-shaped beam, which houses the FBG strain sensor. The schematics of the sensor and the rhombus mechanism employed in this work are shown in Figure 1. The strain sensor is attached to the test article via the input terminals of the rhombus mechanism. The motion of the input terminals is attenuated at the output terminals of the mechanism and transferred to the motion of the ends of the sensing beam. In this configuration, the sensing segment of beam is subjected to pure bending load in addition to the tensile or compressive force. The mechanism attenuates the input displacement and amplifies the input force that is transferred to the beam.

In order to relate the input displacement to the strain developed on the surface of the sensing beam, it is necessary to know the relationship between displacements and forces at input and output terminals of the mechanism.

### Analytical Model

**Figure 2:**
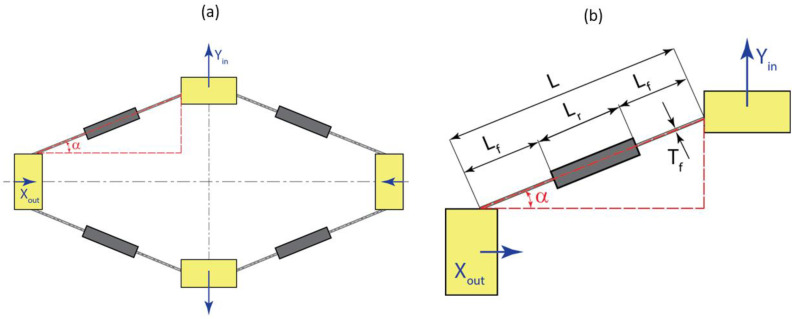
Geometry of the rhombus mechanism: (**a**) View of the full mechanism; (**b**) Quarter-mechanism and the corresponding geometric parameters.

In this work we adopt the modeling approach for a rhombus-type mechanism presented by Li et al. [37]. The approach is based on representation of the generalized beam flexures using the beam constraint models developed by Awtar et al. [38] Utilizing symmetry for simplification of the analysis, we consider a quarter of the rhombus mechanism as shown in Figure 2.

**Figure 3:**
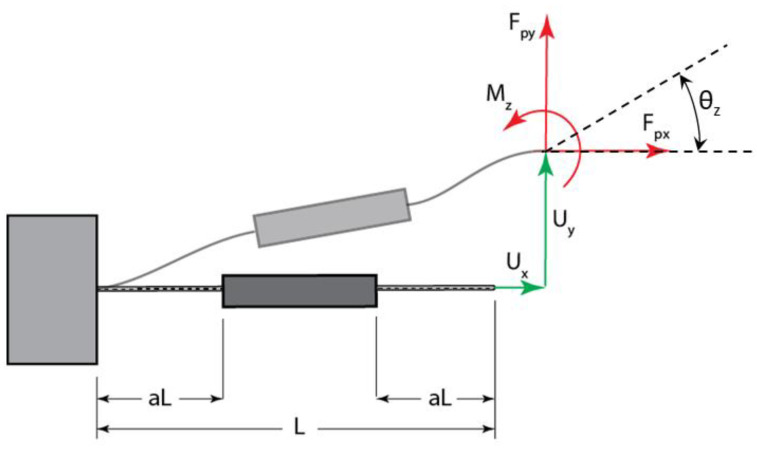
Beam flexure with distributed compliance.

This represents a beam flexure with distributed compliance, as shown in Figure 3. The flexure consists of two flexible segments of length $L_{f}=aL$, where parameter *a* describes the extent of compliance and *L* is the total length of the flexure. The thickness of flexible segments is $T_{f}$. The middle segment of the flexure is considered to be thick enough so that it can be treated as rigid. In order to facilitate analysis, it is convenient to work with non-dimensional forces, moments, and displacements [37]:

(1) $$m_{z}=\frac{M_{z}L}{EI},\;f_{px}=\frac{F_{px}L^{2}}{EI}\;,f_{py}=\frac{F_{py}L^{2}}{EI},\;u_{x}=\frac{U_{x}}{L},\;u_{y}=\frac{U_{y}}{L}$$

where *E* is the modulus of elasticity, I is the second moment of area of the cross-section, $U_{x}$ and $U_{y}$ are axial and transverse displacements of the tip in the local coordinates aligned with the axis of the flexure, and *θ_z_* is the slope at the end of the flexure. According to [37,38], the bending and axial deformations for the generalized flexure are expressed as:

(2) $$\left[\begin{array}{c} f_{py}\\ m_{z} \end{array}\right]=\left[\begin{array}{cc} k_{11}^{0} & k_{12}^{0}\\ k_{21}^{0} & k_{22}^{0} \end{array}\right]\left[\begin{array}{c} u_{y}\\ \theta_{z} \end{array}\right]+f_{x}\left[\begin{array}{cc} k_{11}^{1} & k_{12}^{1}\\ k_{21}^{1} & k_{22}^{1} \end{array}\right]\left[\begin{array}{c} u_{y}\\ \theta_{z} \end{array}\right],$$

(3) $$u_{x}=\frac{f_{px}}{k_{33}}+\left[\begin{array}{cc} u_{y} & \theta_{z} \end{array}\right]\left[\begin{array}{cc} g_{11}^{0} & g_{12}^{0}\\ g_{21}^{0} & g_{22}^{0} \end{array}\right]\left[\begin{array}{c} u_{y}\\ \theta_{z} \end{array}\right]+f_{px}\left[\begin{array}{cc} u_{y} & \theta_{z} \end{array}\right]\left[\begin{array}{cc} g_{11}^{1} & g_{12}^{1}\\ g_{21}^{1} & g_{22}^{1} \end{array}\right]\left[\begin{array}{c} u_{y}\\ \theta_{z} \end{array}\right],$$

where $k_{33}=\frac{6{\left(\frac{L}{T_{f}}\right)}^{2}}{a}$, and the coefficients *k* and *g* were originally derived in [38], and are provided here for convenience in the Appendix A. The ends of the flexure are fixed, hence the angular displacement (slope) $\theta_{z}$ is zero, which simplifies Equations (2) and (3) to [37]:

(4) $$f_{py}=k_{11}^{0}u_{y}+f_{px}k_{11}^{1}u_{y}$$

(5) $$u_{x}=\frac{f_{px}}{k_{33}}+u_{y}{}^{2}g_{11}^{0}+f_{px}u_{y}{}^{2}g_{11}^{1}$$

**Figure 4:**
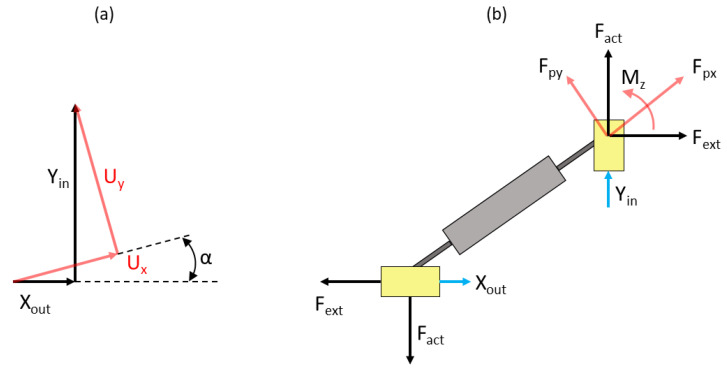
Relationships between displacements and forces at input and output terminals of the rhombus mechanism: (**a**) Schematic for coordinate transformation between local and global for axial and transverse displacements of the tip; (**b**) Force and moment diagram for the quarter-mechanism.

The input terminal will be activated by an input force *F_act_*, and the output terminal will experience a resistive force *F_ext_* due to the presence of the U-shaped sensing beam with the FBG sensor. The schematic of relationships between displacements and forces acting on the terminals of the rhombus mechanism is shown in Figure 4.

This allows us to develop the following transformations between displacements and forces in local and global coordinates [37]:

(6) $$\left[\begin{array}{c} X_{out}\\ Y_{in} \end{array}\right]=\left[\begin{array}{cc} \cos\alpha & -\sin\alpha \\ \sin\alpha & \cos\alpha \end{array}\right]\left[\begin{array}{c} U_{x}\\ U_{y} \end{array}\right]$$

(7) $$\left[\begin{array}{c} F_{px}\\ F_{py} \end{array}\right]=\left[\begin{array}{cc} \sin\alpha & \cos\alpha \\ \cos\alpha & -\sin\alpha \end{array}\right]\left[\begin{array}{c} F_{act}\\ F_{ext} \end{array}\right]$$

Note that in the relationships provided above, *F_act_* represents half of the actual force applied to the rhombus input terminal, as it is assumed that the input force will be divided equally between both sides of the rhombus. Hence, given the input force *F_act_* and the resistive force *F_ext_*, one can use Equations (1) and (7) to compute forces *f_px_* and *f_py_*, then determine *u_x_* from Equation (5) and *u_y_* from Equation (4) as:

(8) $$u_{y}=\frac{f_{py}}{k_{11}^{0}+f_{px}k_{11}^{1}}$$

Having obtained deformations *u_x_* and *u_y_*, it is possible to compute displacements *Y_in_* and *X_ou_*_t_ using Equations (1) and (6). The resistive force *F_ext_* will depend upon the deformation and geometry of the sensing beam mounted on the rhombus mechanism. We proceed with analysis of the U-shaped sensing beam, the ends of which will undergo displacements *X_out_*.

**Figure 5:**
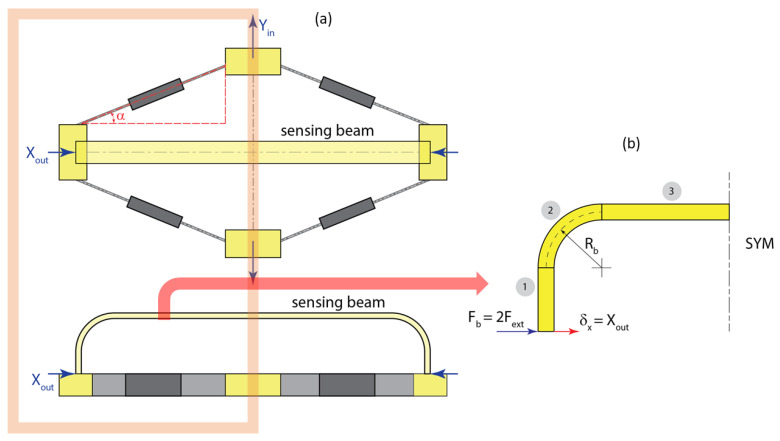
U-shaped sensing beam attached to the rhombus mechanism: (**a**) Front and top views of the mechanism and the sensing beam; (**b**) Schematic of the half-beam used in the model: (1)—vertical segment, (2)—curved segment, (3)—horizontal segment.

As shown in Figure 5a, the ends of the sensing beam are attached to the output terminals of the rhombus mechanism and undergo the same displacement *X_ou_*_t_. We can consider half of the sensor structure for analysis due to symmetry. The goal here is to develop a relationship between the displacement at the ends of the beam mounted at the output terminals of the mechanism and the strain generated at the location of the FBG sensor mounted on the upper surface of the beam. In addition to that, it is necessary to determine the counteracting force *F_ext_* generated by the beam due to that deformation. Since we considered a quarter of the rhombus mechanism for computing its displacements, the force acting at each end of the beam is *F_b_ =* 2*F_ext_*. The relationship between the force *F_b_* and displacement *δ_x_ = X_out_* at the end of the beam can be obtained via Castigliano’s theorem.

Displacement *δ_x_* is related to the strain energy stored in the beam according to:

(9) $$\delta_{x}=\frac{\partial U}{\partial F_{b}}=\frac{\partial \left(U_{1}+U_{2}+U_{3}\right)}{\partial F_{b}}\;,$$

where *U*_1_, *U*_2_, and *U*_3_ are the terms representing the strain energy stored in the three segments of the sensing beam, as shown in Figure 5b. The strain energy in the first (vertical) segment will contain contributions from bending and transverse shear:

(10) $$U_{1}=U_{1}^{b}+U_{1}^{ts}=\overset{L_{1}}{\underset{0}{\int }}\frac{M_{1}^{2}}{2EI}ds+\overset{L_{1}}{\underset{0}{\int }}\frac{cV_{1}^{2}}{2AG}ds=\overset{L_{1}}{\underset{0}{\int }}\frac{F_{b}^{2}s^{2}}{2EI}ds+\overset{L_{1}}{\underset{0}{\int }}\frac{cF_{b}^{2}}{2AG}ds,$$

where *A* is the area of the cross-section, *E* is the elastic modulus, *G* is the shear modulus, *I* is the second moment of area, and *c* = 1.2 is the transverse shear correction coefficient for rectangular cross-section. Strain energy stored in the curved segment can be expressed as:

(11) $$U_{2}=\overset{\frac{\pi }{2}}{\underset{0}{\int }}\frac{M_{2}^{2}}{2AEe}d\theta +\overset{\frac{\pi }{2}}{\underset{0}{\int }}\frac{c{\left(F_{b}\cos\theta \right)}^{2}}{2AG}R_{b}d\theta +\overset{\frac{\pi }{2}}{\underset{0}{\int }}\frac{{\left(F_{b}\sin\theta \right)}^{2}}{2AE}R_{b}d-\overset{\frac{\pi }{2}}{\underset{0}{\int }}\frac{M_{2}\left(F_{b}\sin\theta \right)}{2AE}d\theta ,$$

where *R_b_* is the centroidal radius, $M_{2}=F_{b}\left(L_{1}+R_{b}\sin\theta \right)$, and the eccentricity is computed as:

(12) $$e=R_{b}-\frac{h_{b}}{\ln\left(\frac{R_{b}+0.5h_{b}}{R_{b}-0.5h_{b}}\right)}\;,$$

where *h_b_* is the thickness of the beam cross-section. The strain energy stored in the horizontal segment of the beam is expressed as:

(13) $$U_{3}=\overset{L_{3}}{\underset{0}{\int }}\frac{{\left(M_{2}^{\prime }\right)}^{2}}{2EI}ds+\overset{L_{3}}{\underset{0}{\int }}\frac{F_{b}^{2}}{2AE}ds,$$

where $M_{2}^{\prime }=F_{b}\left(L_{1}+R_{b}\right)$. Integrating and substituting in Equation (9), we obtain:

(14) $$\delta_{x}=F_{b}\left[\frac{L_{1}^{3}}{3EI}+\frac{cL_{1}}{AG}+\left(\frac{0.5\pi L_{1}^{2}+2L_{1}R_{b}+0.25\pi R_{b}^{2}}{2AEe}+\frac{\pi R_{b}}{4AE}-\frac{4L_{1}+\pi R_{b}}{2AE}+\frac{c\pi R_{b}}{4AG}\right)+\frac{L_{3}{\left(L_{1}+R_{b}\right)}^{2}}{EI}+\frac{L_{3}}{AE}\right]$$

**Figure 6:**
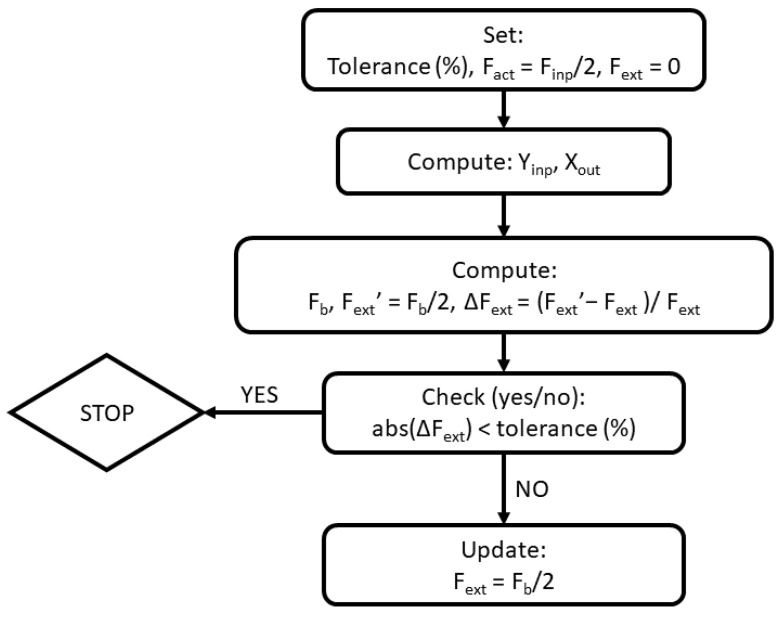
Iterative procedure for computing response of the sensor.

Equation (14) allows us to compute force *F_b_* given displacement *δ_x_* at the end of the sensing beam. Due to interdependency of deformations and forces in the sensor structure, we adopt an iterative procedure for computing the response of the sensor. The schematic of the procedure is presented in Figure 6. Computations typically converge after only 3–4 iterations so the computational cost is very low.

**Figure 7:**
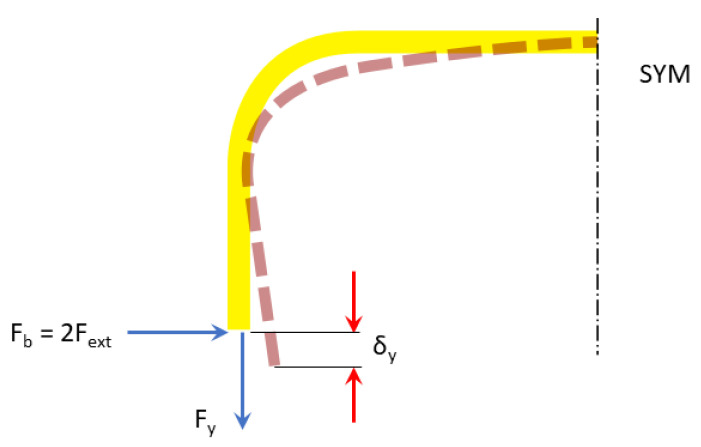
Vertical deformation of the sensing beam: solid yellow—undeformed configuration, dashed brown—deformed configuration.

The sensing beam will undergo relatively large deformations, causing geometric nonlinearity. Therefore, it is necessary to compute its vertical deformation at the location of the FBG sensor, which affects the bending moment and, consequently, the strain at that location. The horizontal segment of the beam is by far the largest contributor to this deformation; hence, we only consider this segment to simplify the analysis. We use the Castigliano’s theorem and apply a fictitious force *F_y_* in the vertical direction at the end of the sensing beam connected to the output terminal of the rhombus mechanism, as shown in Figure 7.

The strain energy stored in the third (horizontal) segment then becomes:

(15) $$U_{3}=\overset{L_{3}}{\underset{0}{\int }}\frac{{\left(M_{2}^{\prime \prime }\right)}^{2}}{2EI}ds+\overset{L_{3}}{\underset{0}{\int }}\frac{F_{b}^{2}}{2AE}ds,$$

where $M_{2}^{\prime \prime }=F_{b}\left(L_{1}+R_{b}\right)+F_{y}\left(s+R_{b}\right)$. Integrating and substituting into $\delta_{y}={\left.\frac{\partial U_{3}}{\partial F_{y}}\right|}_{F_{y}=0}$ yields:

(16) $$\delta_{y}=\frac{F_{b}\left(L_{1}+R_{b}\right)\left(L_{3}^{2}+2L_{3}R_{b}\right)}{2EI}$$

Finally, the strain on the top surface of the sensing beam is computed as:

(17) $$\epsilon_{xx}=\frac{0.5F_{b}\left(L_{1}+R_{b}+\delta_{y}\right)h_{b}}{EI}-\frac{F_{b}}{AE}$$

We now proceed to verification of the developed analytical model via finite element modeling and present experimental results.

## 3. Results

### 3.1. Verification with Finite Element Analysis

**Figure 8:**
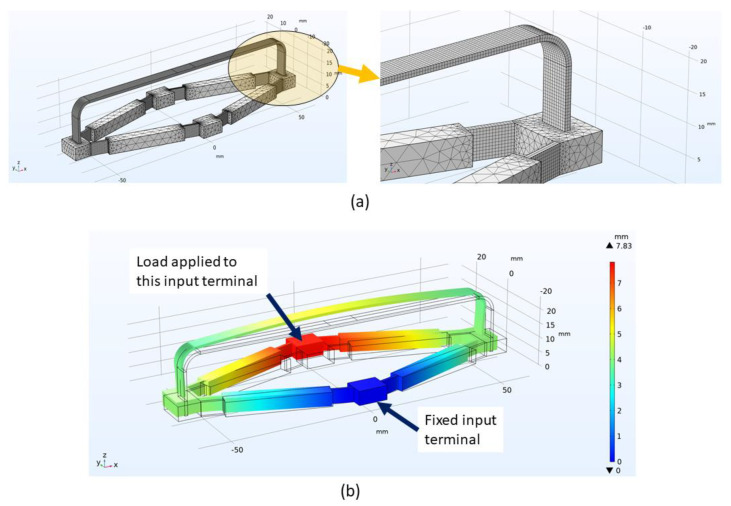
Details of the finite element model: (**a**) Details of the mesh; (**b**) Deformed (color) and undeformed (wireframe) geometry of the modeled sensor under extensional load.

In order to verify the derived analytical model, we employed finite element models built using COMSOL Multiphysics commercial software with the Structural Mechanics module. Several realizations of possible sensor geometries were considered with *L* = 50 and 25 mm, *a* = 0.15 and 0.35, and angle *α* = 10° and 15°. The material of the rhombus mechanism and the sensing beam was considered to be a generic ABS plastic with an elastic modulus *E* = 2.0 GPa and Poisson’s ratio *ν* = 0.35. The model utilized elements with quadratic Lagrange discretization. The sensing beam and flexible hinges of the mechanism were meshed using mapped meshing, while the parts undergoing mostly rigid body motions were meshed with tetrahedral elements. Details of the mesh are provided in Figure 8a. One of the input terminals of the mechanism was fixed, while the input force was applied to the other terminal in the direction of the line between two input terminals as shown in Figure 8b. The finite element analysis was run, including the geometric nonlinearity.

**Figure 9:**
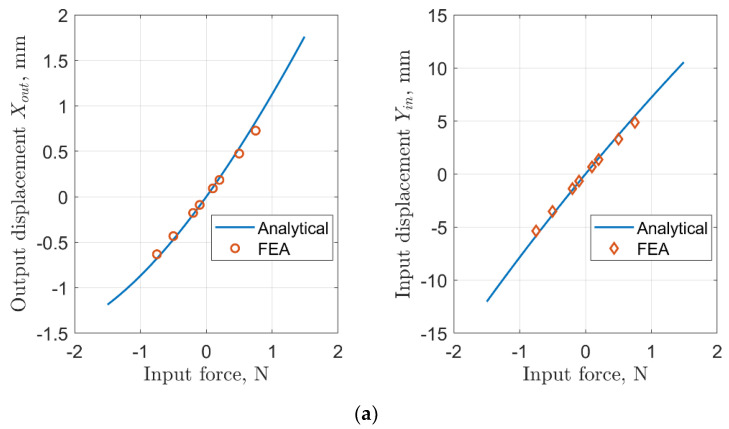

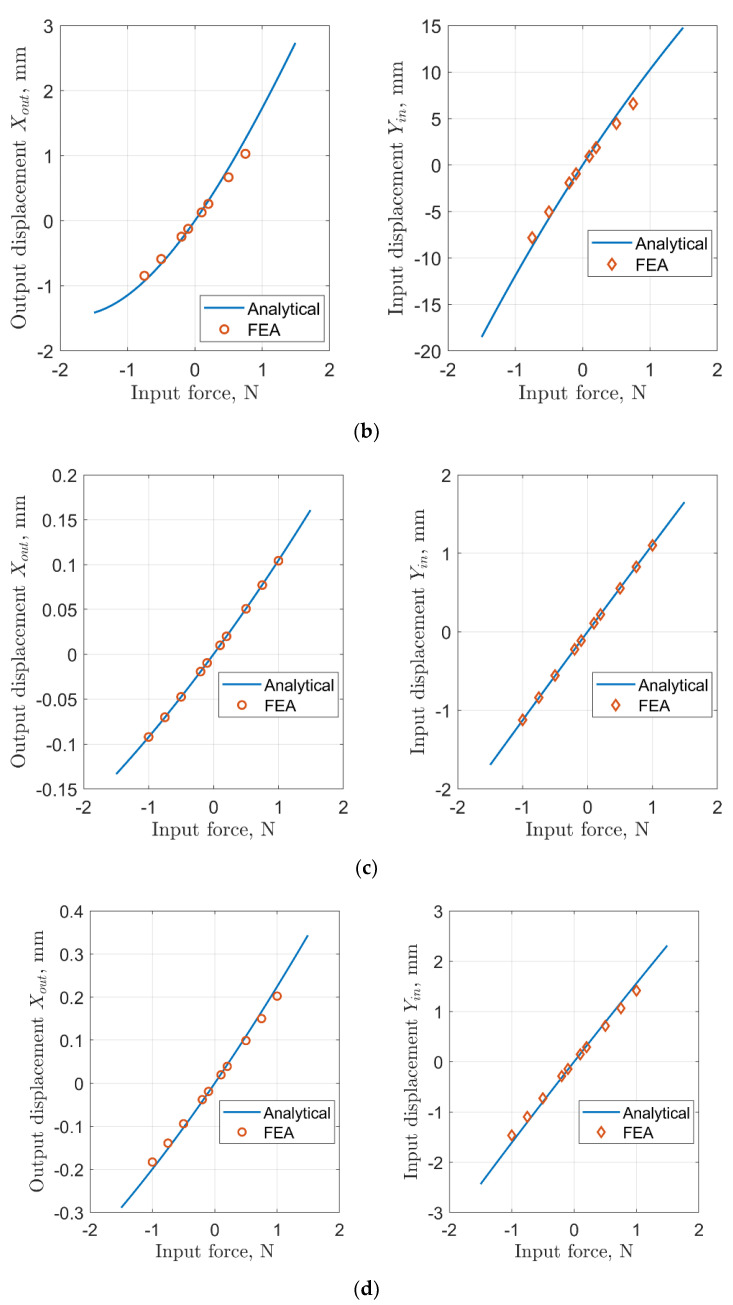
Input and output displacements at the terminals of the rhombus mechanism: (**a**) Case 1: *L* = 50 mm, *a* = 0.15, *α* = 15°; (**b**) Case 2: *L* = 50 mm, *a* = 0.35, α = 15°; (**c**) Case 3: *L* = 25 mm, *a* = 0.15, *α* = 10°; (**d**) Case 4: *L* = 25 mm, *a* = 0.35, *α* = 15°.

First, we compare input and output displacements at the terminals of the mechanism between analytical and finite element models in Figure 9. One can clearly observe that predictions from both models are in reasonably good agreement.

**Figure 10:**
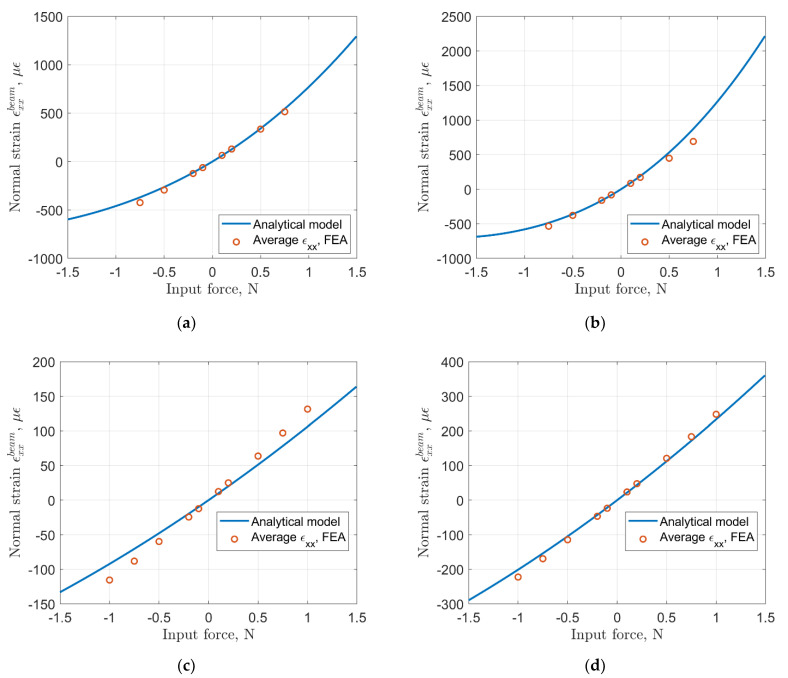
Normal strain on the sensing beam: (**a**) Case 1: *L* = 50 mm, *a* = 0.15, *α* = 15°; (**b**) Case 2: *L* = 50 mm, *a* = 0.35, *α* = 15°; (**c**) Case 3: *L* = 25 mm, *a* = 0.15, *α* = 10°; (**d**) Case 4: *L* = 25 mm, *a* = 0.35, *α* = 15°.

We also compared normal strain predicted by the analytical model and the average strain from the finite element model at the top surface of the sensing beam. Results are shown in Figure 10.

It is evident that for longer flexure (larger *L*), the relationship between the input force and the output strain is strongly nonlinear. Shorter flexures result in an input force–output strain relationship that is much closer to the linear one. One can observe that the analytical model and the FE model are generally in good agreement, with somewhat larger discrepancies for case 3, which combines shorter flexures with smaller compliant segments (*a* = 0.15) and a smaller angle *α*. Next, we discuss the experimental validation of the analytical model.

### 3.2. Experimental Validation

Sensor prototypes were manufactured using the Stratasys Objet260 Connex3 3D printer and the RGD-5131-DM material that simulates engineering plastics. The material was characterized to determine the modulus of elasticity in bending via a 3-point beam bending test on an MTS servo-hydraulic test frame following ASTM D790-17 standard [39]. The obtained value of the elasticity modulus in bending was 2.406 ± 0.019 GPa. This value was subsequently used in the analytical model for comparing its predictions with experimental results.

The rhombus mechanisms and sensing beams were printed separately and then assembled and joined using an epoxy adhesive. The prototypes have the following geometric parameters: rhombus arm length *L* = 50 mm, angle *α* = 15°, and parameter *a* = 0.15. The thickness of the sensing beam is 1 mm, its width is 5 mm, the length of vertical segments is 10 mm, the centroidal radius of the curved segment is *R_b_* = 5 mm, and the length of the horizontal segment is 103.5 mm. We employed T10 FBG strain sensors from Technica Optical Components. These sensors are based on a standard single mode fiber with polyimide coating and a gauge length of 10 mm. Strain and temperature sensitivity are quoted by the supplier as 1.2 pm/µε and 10 pm/°C. The FBG gauges were glued onto the sensing beams of both prototypes, using a cyanoacrylate adhesive such that the gauge was located at the midspan of the sensing beam.

**Figure 11:**
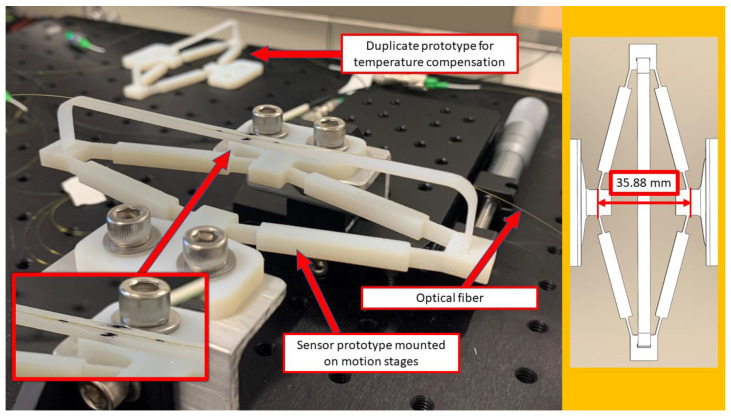
Photo of the protype mounted on motion stages.

One of the prototypes was carefully aligned and mounted on two Thorlabs XR-50P linear translation stages. The other prototype was set aside in the immediate vicinity to be used for compensation of the strain changes due to thermal effects. LUNA Hyperion si155 optical interrogator with ENLIGHT software was used to collect the data from FBG strain sensors. The experimental prototype mounted on the translation stages is shown in Figure 11.

**Figure 12:**
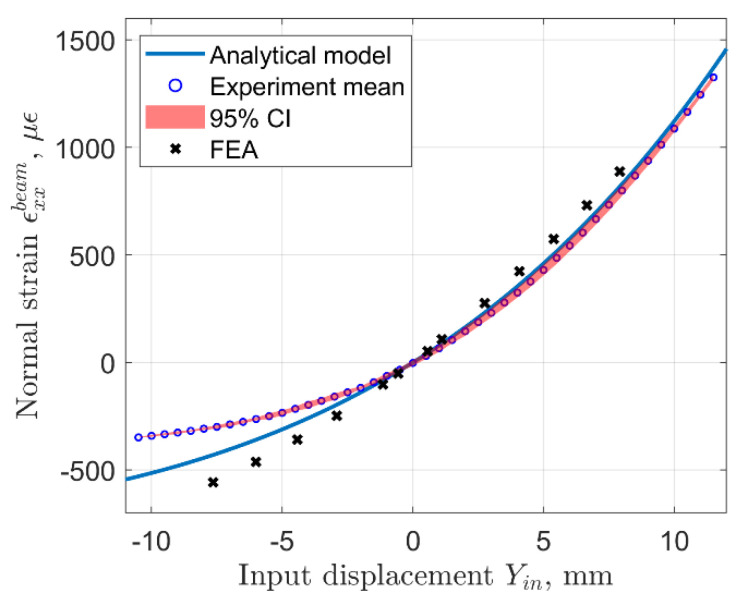
Input displacement vs. strain measured on the sensing beam, analytical prediction-solid line, experimental data-dotted line.

**Figure 13:**
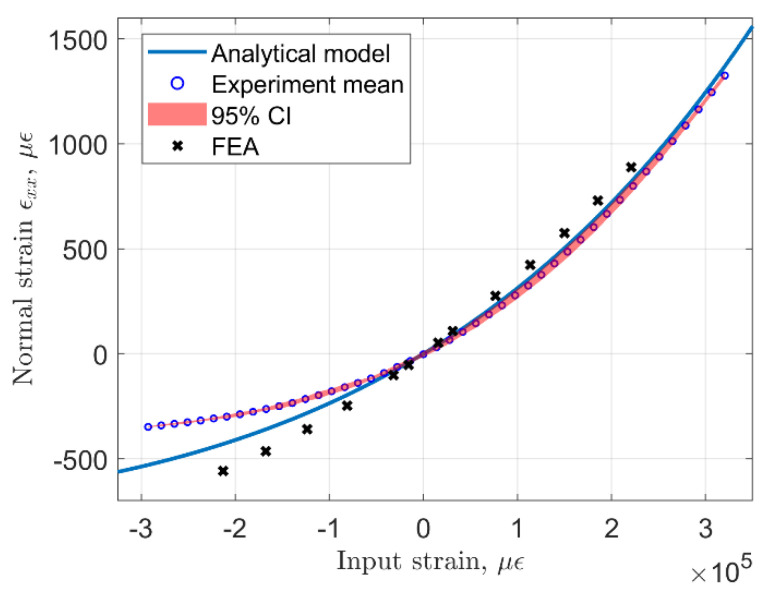
Input strain vs. strain measured on the sensing beam, analytical prediction-solid line, experimental data-dotted line.

During the experiment, the distance between the input terminals was changed manually, using linear translation stages with an accuracy of ±0.02 mm. The origin for the measurement of input displacement was set using a caliper to represent the undeformed configuration of the sensor based on its 3D model. In practice, this could be done using a template for the specific sensor geometry which will depend on the rhombus arm length and the angle. The collected experimental data is compared with predictions from the analytical model and FEA results in Figure 12 and Figure 13. Here, both the analytical and FE models utilized the experimentally obtained value of elastic modulus (2.406 ± 0.019 GPa.) We show the mean value and the 95% confidence interval obtained from four full loading and unloading cycles performed during the experiment.

The input strain in Figure 13 is computed as the ratio of input displacement to the distance between the centers of the input terminals, which in this case was 35.88 mm. We must note that only the input terminals of the mechanism should be glued to the test article, while the output terminals should not be adhered to the test article. From the data plotted in these figures, one may observe that the analytical model prediction is very close to the experimental data in the positive range of input displacements, which result in positive strain measured on the sensing beam. In the negative range of input displacements (compression of the sensor), the analytical model predicts higher magnitude of strain than what is observed in the experiment and the difference between them increases for larger compressive input strains. Strain obtained from the FEA model of the experimental prototype appears to have a larger discrepancy with experimental data than the analytical model.

Note that the sensor response is nonlinear and is similar to what is depicted in Figure 12 because the geometric configuration is the same. Ideally, it is desirable to have a sensor with a linear response through the entire operational range for simplicity of data, but in this case nonlinear calibration will be necessary. It is possible to fit a quadratic polynomial to the experimental data using the least squared error approach, which results in the following expression: $\epsilon_{xx-exp}^{beam}=3.814Y_{inp}^{2}+69.67Y_{inp}+0.04137$, where *Y_inp_* is the input displacement in mm, and *R*^2^ = 0.9993. Similarly, the analytical model can also be very well fitted with a quadratic function $\epsilon_{xx-an}^{beam}=2.928Y_{inp}^{2}+81.92Y_{inp}+2.399$, and *R*^2^ = 0.9988. Overall, it can be stated that the analytical model provides fairly accurate predictions of the sensor response, particularly for the extensional range of sensor input motion.

**Figure 14:**
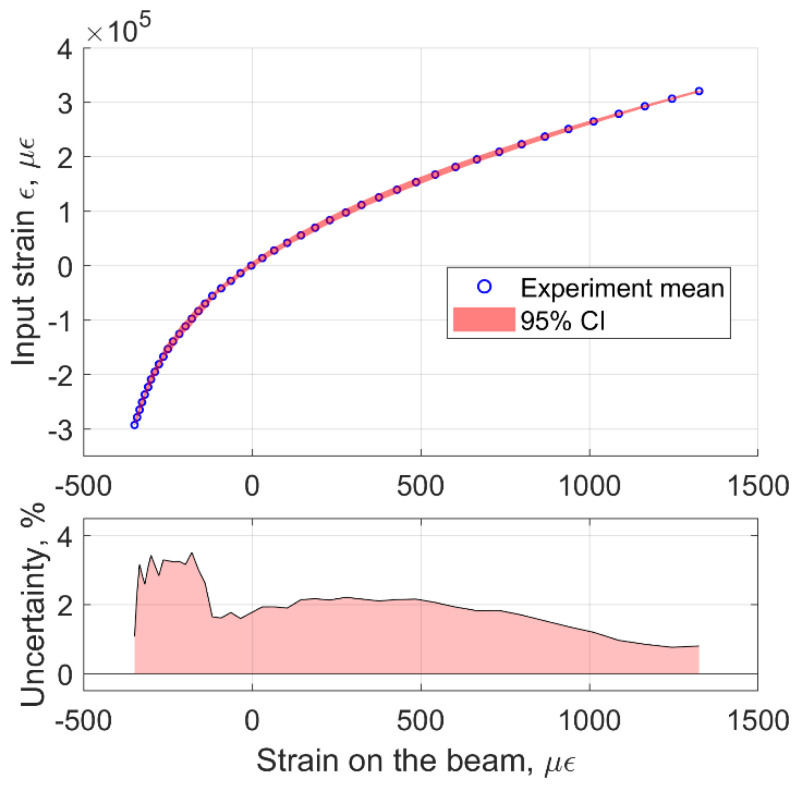
Mapping of the strain measured on the beam to the strain applied at the input terminals including the measured uncertainty.

In addition, we performed error analysis on to determine the amount of uncertainty when the strain measured on the beam is mapped back to determine the strain applied at the input terminals. Results are plotted in Figure 14. The maximum amount of uncertainty in the measured input strain is determined to be 3.51% with respect to the total range of input strain.

It is important to note that the strain transferred from the sensing beam to the FBG sensor is affected by the bonding geometry and material properties of the beam, the optical fiber and its coating, and the adhesive. In practice, if bonding is performed manually, it is difficult to control the bonding thickness and other parameters with very tight accuracy. According to studies, the strain transfer rate can be about 70–80% [18,40,41]. However, determination of the exact value of the strain transfer rate is not important for this sensor design. The simple reason for this is that for each manufactured sensor, its strain output must be calibrated to the input strain to obtain the resulting mapping function. In addition, this is necessary for the purpose of being able to backout the input strain/displacement from the strain measured by the FBG gauge on the sensing beam. This calibration process takes care of variability in the strain transfer rates from the sensing beam to the FBG.

In order to implement compensation of thermally induced strains, it is necessary to include an additional FBG on an unloaded part of the structure, for example on the top surface of one of the output terminals of the rhombus mechanism. Alternatively, thermal effects could be compensated by placing an additional FBG on the bottom surface of the sensing beam, which will constitute a typical configuration commonly used for measuring bending strains with thermal compensation. We discuss the effects of geometric design parameters on the behavior of the sensor next.

## 4. Discussion

**Figure 15:**
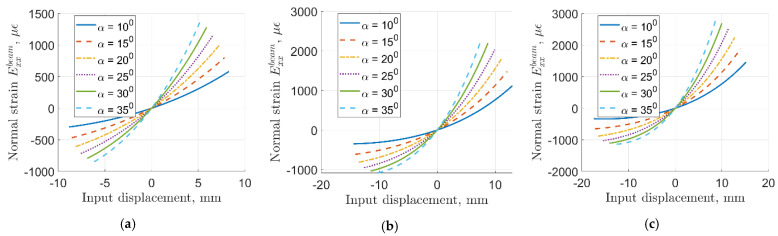
Strain response prediction for different mechanism geometries: (**a**) a = 0.1; (**b**) a = 0.2; (**c**) a = 0.3.

Here, we consider the effects of the rhombus angle *α* and the parameter *a* on the sensor behavior. For the sake of this discussion, we leave all properties and other parameters (e.g., geometry of the sensing beam) the same as what has been used for the experimental prototypes. The range of input force values in the analytical model was between −1.75 N to 1.75 N in all cases presented here. The plots of the resulting strain on the sensing beam are plotted versus the corresponding input displacements in Figure 15. From the data plotted in these figures, it is clear that lower values of parameter *a* result in stiffening of the sensor, as evident from smaller range of input displacements for *a* = 0.1 compared to *a* = 0.3. The range of resulting strains with *a* = 0.1 is significantly smaller (almost twice as small) compared to cases with *a* = 0.3.

Angle *α* mostly affects strain attenuation, as evidenced from changes in the slope of the curves plotted in these figures. Higher values of angle *α* result in lower compliance of the mechanism, reduced attenuation ratio, and larger strain on the sensing beam. It needs to be mentioned that larger values of parameter *a* result in longer flexible segments of the rhombus arms, thus resulting in higher compliance of the mechanism and stronger nonlinearity of the input–output strain relationship. Reducing the arm length values will generally result in lower compliance, but higher linearity of the sensor behavior is evident from Figure 10. The design of the sensor based on the rhombus compliant mechanism shall be tailored to allow the required range of input strain, external dimensions, and material to fit a particular application.

## 5. Conclusions

In this work we proposed and evaluated a fiber optic-based sensor for measuring large strains that utilizes a rhombus-type compliant mechanism. The purpose of the rhombus mechanism is to attenuate the input motion and transfer it to the ends of sensing beam with the mounted FBG strain gauge. We developed an analytical model that allows one to quickly and easily predict performance of a sensor design given specific values of geometric parameters and hence optimize the design for a particular application, e.g., input strain/displacement range, desired dimensions, etc. The prototype manufactured and tested in this work was able to handle input strains exceeding ±2.5 × 10^5^ µε with measurement uncertainty of approximately 3.5%.

The disadvantage of the proposed sensor is that its input–output strain characteristic is nonlinear due to inherent kinematics of the rhombus mechanism. Hence, each sensor will require careful calibration prior to being deployed in an application. The advantage of the proposed sensor design is that it allows us to reduce the stiffening effect and the force feedback from the sensor onto the structure being tested. The main possible application area for this type of sensors would be situations where the structure under test is very compliant, experiences large quasi-static strains, and direct adhesion of an FBG strain gauge is impossible due to stiffness mismatch between the optical fiber and the structure. This is particularly relevant to parts and devices made of rubber or shape-memory polymers and has potential applications in soft robotics and prosthetics.

## Data Availability

The data presented in this study are available on request from the corresponding author.

## Appendix A

Coefficient matrices for the terms in Equations (2) and (3):

(A1) $$\left[\begin{array}{cc} k_{11}^{0} & k_{12}^{0}\\ k_{21}^{0} & k_{22}^{0} \end{array}\right]=\frac{1}{a\left(3-6a+4a^{2}\right)}\left[\begin{array}{cc} 6 & -3\\ -3 & 3-6a+4a^{2} \end{array}\right]$$

(A2) $$\left[\begin{array}{cc} k_{11}^{1} & k_{12}^{1}\\ k_{21}^{1} & k_{22}^{1} \end{array}\right]=\frac{1}{5{\left(3-6a+4a^{2}\right)}^{2}}\left[\begin{array}{cc} 3\left(15-50a+60a^{2}-24a^{3}\right) & -a\left(15-60a+84a^{2}-40a^{3}\right)\\ -a\left(15-60a+84a^{2}-40a^{3}\right) & a\left(15-60a+92a^{2}-60a^{3}+\frac{40}{3}a^{4}\right) \end{array}\right]$$

(A3) $$\left[\begin{array}{cc} g_{11}^{0} & g_{12}^{0}\\ g_{21}^{0} & g_{22}^{0} \end{array}\right]=\frac{1}{10{\left(3-6a+4a^{2}\right)}^{2}}\left[\begin{array}{cc} -3\left(15-50a+60a^{2}-24a^{3}\right) & a\left(15-60a+84a^{2}-40a^{3}\right)\\ a\left(15-60a+84a^{2}-40a^{3}\right) & -a\left(15-60a+92a^{2}-60a^{3}+\frac{40}{3}a^{4}\right) \end{array}\right]$$

(A4) $$\left[\begin{array}{cc} g_{11}^{1} & g_{12}^{1}\\ g_{21}^{1} & g_{22}^{1} \end{array}\right]=\frac{a^{3}}{175{\left(3-6a+4a^{2}\right)}^{3}}\;\left[\begin{array}{cc} 2\left(105-630a+1440a^{2}-1480a^{3}+576a^{4}\right) & -\left(105-630a+1440a^{2}-1480a^{3}+576a^{4}\right)\\ -\left(105-630a+1440a^{2}-1480a^{3}+576a^{4}\right) & \left(105-630a+1560a^{2}-2000a^{3}+1408a^{4}-560a^{5}+\frac{1120}{9}a^{6}\right) \end{array}\right]$$
